# Transgender individuals are at higher risk for suicidal ideation and preparation than cisgender individuals in substance use treatment

**DOI:** 10.3389/fpsyt.2023.1225673

**Published:** 2023-09-13

**Authors:** Martin Hochheimer, Jennifer L. Glick, Henri Garrison-Desany, Andrew S. Huhn

**Affiliations:** ^1^Department of Psychiatry and Behavioral Sciences, School of Medicine, Johns Hopkins University, Baltimore, MD, United States; ^2^Department of Health, Behavior, and Society, Johns Hopkins Bloomberg School of Public Health, Baltimore, MD, United States; ^3^Department of Social and Behavioral Science, Harvard University T.H. Chan School of Public Health, Boston, MA, United States

**Keywords:** substance use disorder, gender identity, transgender, suicide, treatment retention, propensity score matching

## Abstract

**Introduction:**

This study describes the differences and similarities in mental health, substance use, and substance use treatment outcomes between people presenting for SUD treatment who identified as transgender and those who identified as cisgender men or women.

**Methods:**

We compared 64 individuals who self-identified as transgender and presented for SUD treatment to samples of cisgender men and women (separately) matched based on propensity scores which were created based on sociodemographic factors known to influence both the nature of substance use and patterns of treatment engagement including age, education, race, stable housing, and employment status. Comparisons were made using χ^2^ tests and *t-*tests in over 150 variables collected at treatment intake regarding physical and mental health, substance use patterns, events that led to treatment, reasons for seeking treatment, and treatment outcomes.

**Results:**

The transgender sample endorsed six of the seven suicide-related items more often than at least one of the cisgender-matched samples. Furthermore, the transgender sample remained in treatment significantly longer (M = 32.3, SD = 22.2) than the cisgender male sample (M = 19.5, SD = 26.1, t = 2.17, *p* = 0.03).

**Discussion:**

This study is a first step into understanding gender minority population experiences during SUD treatment. While there was no significant difference between the cisgender and transgender samples on most variables, there was an elevated prevalence of suicidal ideation and behaviors in the transgender sample, which warrants further investigation.

## 1. Introduction

Gender minority populations have an increased risk of developing substance use disorders (SUDs) (1) and other mental health disorders, including major depressive disorder, anxiety disorders, personality disorders, and suicide-related outcomes (2) compared with the general US population. However, gender identity is rarely reported in substance use studies (3). A 2022 scoping review indicated that there were no studies that investigated the differences in substance use treatment intervention outcomes that compared gender minorities to cisgender samples (i.e., samples of individuals whose gender identity corresponds to their sex assigned at birth) (4). The lack of inclusion of gender minority populations stymies the ability of SUD treatment providers to identify and address any specific needs that may exist in these populations.

To understand gender minority individuals, language and conceptualizations are constantly evolving, and measurement of best practices is still emerging (5–7). The term “transgender” encompasses a wide range of gender minority populations and can include persons whose gender identity is different from the sex they were assigned at birth, such as those with non-binary identities (6). The minority stress model explains that the marginalization, discrimination, and stigmatization of minority groups, such as transgender populations, by the majority leads to specific patterns of substance use and mental health disorders, which differ from that of cisgender individuals (8–11). It is necessary to explore if these patterns of marginalization also change the SUD treatment experience and trajectory of transgender individuals.

To better tailor interventions to individuals in substance use treatment, it is necessary to understand the differences between individuals of different genders. This information can be used to develop and establish culturally appropriate care since the current treatment protocols were primarily developed for cisgender heterosexual men and are applied to all without regard for gender (12). It is easier, quicker, and more common for treatment providers to modify existing treatment practices than create them from scratch—regardless of the appropriateness of such an approach. Knowing if and how transgender individuals differ from cisgender individuals when entering treatment, as well as their similarities and differences in treatment outcomes, is critical to public health endeavors and will facilitate the introduction of more suitable treatments.

Previous research has found that during SUD treatment, there are well-established differences between cisgender men and cisgender women (13). One study found that among individuals in substance use treatment, cisgender women had higher rates of all mood and anxiety disorders than cisgender men, while cisgender men had higher rates of narcissistic and antisocial personality disorders (14). This finding aligns with general population studies, which have found that cisgender women have higher rates of lifetime major depressive disorder and PTSD than cisgender men (15, 16). Less is known about the differences between cisgender and transgender individuals. Though several reviews have not found significant gender differences in treatment outcomes, the reviewers attributed this to a lack of studies that report outcomes by gender or sex (17–20). The dearth of gender difference reporting in SUD treatment studies was highlighted in a 2022 systematic review which found that of the 316 clinical trials for SUD treatment between 2010 and 2019, only 8% provided gender- and/or sex-specific analyses, and only 1.5% reported any transgender participants (18).

Given that previous research has found that gender disparities in mental health exist in the United States (21, 22) and that better practices in gender identity measurement have not yet been widely adopted (CITE- me and others), there is an urgent need to report any findings that may begin to identify clinical targets to improve outcomes for those who identify as transgender. This study aimed to describe different factors affecting mental health and substance use patterns among transgender- vs. cisgender-identifying people who present for SUD treatment. These findings have important implications for supporting transgender participants in SUD treatment and tailoring interventions to their specific behavioral health needs.

## 2. Materials and methods

### 2.1. Sample

Data were collected from a third-party treatment outcome collection provider, Vista Research Group Inc., which contracts with individual facilities to track patient progress during treatment. The parent sample included 38,091 individuals who sought treatment at 86 addiction and mental health treatment centers across the US, including individuals in residential substance use treatment (*n* = 13,669), detoxification programs (*n* = 10,939), partial hospitalization programs (*n* = 4,752), outpatient SUD programs (*n* = 3,169), or broad mental health programs (*n* = 1,835). Standardized assessments were delivered to each individual by their provider through a HIPAA-compliant portal on a computer or tablet. De-identified individual-level data were transferred to the study investigators through a data use agreement; the Johns Hopkins University Institutional Review Board reviewed and acknowledged that this study did not constitute research on human subjects. Data presented here were collected from individuals admitted to treatment from January 2016 to October 2020.

### 2.2. Gender

Each person who presented for treatment was asked whether they identified as male, female, or transgender; 64 individuals identified themselves as transgender. No other information about gender identity or sex assigned at birth was available to further contextualize the sample; therefore, this study chose to use a broad definition of transgender to accommodate the participants' personal interpretation of the options on the intake form.

### 2.3. Intake measures

#### 2.3.1. CAGE

The CAGE is a 4-item questionnaire developed to identify people with alcohol use disorder. The screener asks the individual to answer yes or no if they have ever experienced any of four situations that are a consequence of alcohol use (23), two or more yes answers indicate a clinically significant indication of alcohol use disorder. The scale has been adapted for use in many languages, cultures, and many other substances with high reliability (24–26).

#### 2.3.2. Patient health questionnaire

The PHQ-9 is a 9-item instrument used to screen major depressive disorder in which the patient is asked how often they have experienced specific symptoms of depression (such as feeling down, depressed, or hopeless) in the last 2 weeks and are asked to endorse one of four choices (not at all = 0, several days = 1, more than half the days = 2, or nearly every day = 3). The responses are added, and a score of 20 or more is indicative of major depressive disorder (27). This scale has been found to be reliable and valid in many contexts (28–30).

#### 2.3.3. Generalized anxiety disorder

The GAD-7 is a 7-item questionnaire designed to assess anxiety which asks about specific symptoms of anxiety over the last 2 weeks and uses the same four choices and scoring system as the PHQ-9. Scores of 10 or higher indicate clinically significant levels of anxiety (31). It has been found to have high levels of reliability and validity (32, 33).

#### 2.3.4. PRIME screening for psychosis

The PRIME is a 12-item questionnaire that screens the prodromal phase of schizophrenia that describes the intensity of the positive symptoms of schizophrenia from zero (definitely disagree) to six (definitely agree), with two or more items endorsed as five or six indicating schizophrenia (34).

#### 2.3.5. Altman self-rating mania scale

The ASRM contains five statements, each of which indicates an emotion or behavior associated with mania (e.g., I talk more than usual). The patient is asked to respond to how much the statement is applicable to them from zero (I do not) to four (I am constantly); a score of six or higher indicates the probability of mania (35). Several studies have confirmed the reliability and validity of this scale (36).

#### 2.3.6. PTSD checklist-civilian version

The PCL-C is a 17-item scale that asks the patient about the degree to which they experience symptoms of PTSD (e.g., repeated, disturbing memories, thoughts, or images of a stressful experience) on a scale from 0 (not at all) to 4 (extremely) (37). Only 6 of the 17 items were included in the survey; the total score ranges from 0 to 24.

#### 2.3.7. Eating disorder diagnostic scale

The EDDS is a 22-item questionnaire designed to measure the presence of three eating disorders: anorexia nervosa, bulimia nervosa, and binge eating disorders (38). The questions assess body self-perception, uncontrolled eating, and behaviors done to offset eating (e.g., fasting and vomiting), which are the symptoms of these disorders. Krabbenborg et al. (39) found that the EDDS had good test–retest reliability, internal consistency, and convergent validity with other eating disorder scales.

#### 2.3.8. Columbia suicide-severity rating scale screening version

At intake, suicide risk was assessed using two preliminary questions. First, all individuals were asked a yes/no screening question, “Have you ever done anything, started to do anything, or prepared to do anything to end your life?” Second, they were asked if they endorsed the statement “One of the reasons that I am seeking treatment is because of suicidality.” Regardless of the patients' answers to these questions, all participants were asked the six questions of the Columbia Suicide-Severity Rating Scale screen version (40). The Food and Drug Administration in 2012 made the CSSR the preferred instrument or “gold standard” for measuring suicidal ideation and behavior (41).

#### 2.3.9. Drug use patterns

Every participant was asked to endorse or deny each of the 11 DSM-5 (42) criteria for substance use disorder. They were also asked to identify their primary substance and usage of 14 substances (alcohol, marijuana, amphetamine, cocaine, methamphetamine, hallucinogens, opiates, benzodiazepines, stimulants, heroin/fentanyl, inhalants, club drugs, synthetic drugs, and other drugs) in the 30 days before treatment. The number of criteria and the number of substances endorsed were compared with the results as continuous variables, while in the Supplementary material, differences between the groups were examined for each criterion and substance.

#### 2.3.10. Events before treatment

Patients were presented with a list of reasons for entering treatment that included 11 specific events which commonly lead to substance use treatment entry (family or friends asked or told me to get into treatment, I became scared or upset by the way I was feeling, I became tired of living this way, I was arrested, I was caught driving under the influence, I was hospitalized, I talked about or attempted suicide, I overdosed, I got alcohol poisoning, I was ordered to treatment by the court, and I was involuntarily committed). The total number of reasons endorsed is reported in the Results section, while comparisons for each condition are found in the Supplementary material.

### 2.4. Outcome measures

#### 2.4.1. Treatment retention

Previous research has established that longer treatment engagement has been associated with reduced mortality, drug use, criminal activity, risky sexual behaviors, and improved social functioning; treatment retention is a key indicator of treatment success (43–48). Therefore, days in treatment were analyzed as an outcome measure of interest.

#### 2.4.2. Treatment completion

While longer treatment engagement is associated with positive outcomes, treatment programs are time limited and patients are discharged either due to successful completion of treatment, discharge against medical advice (AMA), or for several other reasons including death, incarceration, or other administrative reasons (e.g., issues due to payment). Therefore, the comparisons were made based on the proportion of those discharged from AMA and those who successfully completed treatment.

### 2.5. Analysis

Propensity score matching was used to create comparison samples for transgender individuals with cisgender women or cisgender men. Samples were matched using sociodemographic factors known to influence the nature of substance use and patterns of treatment engagement including age, education, race (49), stable housing (50), and being employed either full- or part time (51).

The propensity score was estimated using multivariate logistic regression; then, optimal matching identified the most similar subjects among cisgender women compared with transgender individuals, based on the listed covariates without replacement. The same procedure was performed to match those identified as cisgender men with those identified as transgender. One person who identified as transgender was missing information about stable housing and employment and was matched based on missingness; none of the other variables had missing data.

**Figure 1:**
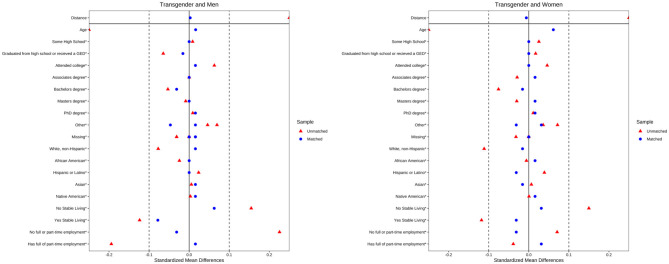
Balance of matched and unmatched samples.

The balance of the resultant samples was tested by ensuring that all variables among cisgender women had a standardized mean difference of <0.1 and variance ratios between 0.5 and 2 (see Figure 1). The cisgender men-matched sample was similarly balanced except for age. The mean age of the transgender sample was 27.03 years (SD 8.67) and that of the cisgender men-matched sample was 29.29 years (SD 9.52), with a standardized mean difference of −0.26. Despite this imbalance, the matched sample was retained for analysis since a Welch two-sample *t*-test was not significant (t = 1.408, df = 124.93, *p* = 0.16), and the effect size was small (Cohen's d = 0.25).

For each analysis, the transgender sample was compared with each cisgender group and reported separately (i.e., transgender people vs. cisgender women; transgender people vs. cisgender men) using two-sample *t-*tests for the means of continuous variables and χ^2^ tests for goodness of fit for categorical variables (α <0.05). Comparisons of effect size have been reported as Cohen's d for comparisons of means and Cohen's ω for categorical variables. Three-group comparisons were not employed because each of the cisgender samples was matched specifically with the transgender sample, precluding the comparison of cisgender men and women.

Using 128 subjects (the transgender sample *n* = 64, and one of the matched cisgender samples, *n* = 64) provides sufficient power (β > 0.80) to detect a medium effect size (Cohen's d = 0.5; Cohen's w = 0.31, df = 4). All analyses were conducted using R software (52), and α levels were set at 0.05.

## 3. Results

The two matched cisgender samples were drawn from a pool of 37,973 individuals, with 64.6% men and 35.4% women. The transgender sample (*n* = 64, 1.7%) was broadly comparable to this initial pool of cisgender individuals with both samples being primarily White (79.2 vs.70.3%) and high school graduates (86.5 vs. 81.25%) who had stable living arrangements (79.3 vs. 67.2%); however, fewer transgender individuals were employed either full-time or part-time (46.9 vs. 64.2%, χ^2^ = 20.65, df = 3, *p* < 0.001) at the start of treatment. The transgender sample was also significantly younger, on average (M = 27.03, SD = 8.67), than the cisgender population (M = 36.22, SD = 12.86, t = −8.46, df = 63.47, *p* < 0.001).

Table 1 displays the demographic makeup of the total sample by gender group and a comparison of the transgender sample to the matched samples of cisgender men and women who were included in the analysis. There was no significant difference in the primary substance used between any of the groups (see Table 2).

**Table 1:** Comparison of demographic/matching and substance use variables.

		**Matched samples** ^**a**^					**Whole cisgender sample** ^**b**^	
**Mean (SD)**		**Transgender**	**Female**		**Male**		**Female**	**Male**
***n*** **(%)**		**(*****n** =* **64)**	**(*****n** =* **64)**		**(*****n** =* **64)**		**(*****n** =* **13,461)**	**(*****n** =* **24,512)**
				**SMD** ^c^		**SMD** ^c^		
Age		27.03 (8.67)	27.44 (9.63)	0.061	29.3 (9.52)	0.003	36.96 (13.15)	35.81 (12.68)
Education	Some High School	6 (9.4%)	10 (15.6%)	0.000	5 (7.8%)	0.016	923 (6.9%)	2,086 (8.5%)
	Graduated HS or GED	16 (25.0)%	17 (26.6%)	0.000	15 (23.4%)	0.000	3,136 (23.3%)	7,712 (31.5%)
	Some College	22 (34.4%)	17 (26.6%)	0.000	22 (34.4%)	−0.016	4,009 (29.8%)	6,887 (28.1%)
	Associate's Degree	5 (7.8%)	2 (3.1%)	0.016	5 (7.8%)	0.000	1,443 (10.7%)	1,913 (7.8%)
	Bachelor's Degree	6 (9.4%)	7 (10.9%)	−0.016	5 (7.8%)	−0.031	2,279 (16.9%)	3,599 (14.7%)
	Master's Degree	2 (3.1%)	1 (1.6%)	0.016	1 (1.6%)	0.000	825 (6.1%)	977 (4.0%)
	Ph.D. Degree	1 (1.6%)	1 (1.6%)	0.016	1 (1.6%)	0.016	62 (0.5%)	161 (0.7%)
	Other	6 (9.4%)	9 (14.1%)	−0.031	10 (15.6%)	0.016	780 (5.8%)	1,172 (4.8%)
Race/Ethnicity	White, non-Hispanic	45 (70.3%)	42 (60.9%)	−0.016	39 (60.9%)	0.016	10,964 (81.5%)	19,128 (78.0%)
	African-American	3 (4.7%)	1 (12.5%)	0.016	8 (12.5%)	0.000	711 (5.3%)	1,746 (7.1%)
	Hispanic or Latino	7 (10.9%)	12 (4.7%)	−0.031	3 (4.7%)	0.000	949 (7.1%)	2,102 (8.6%)
	Asian	1 (1.6%)	1 (3.1%)	−0.016	2 (3.1%)	0.016	123 (0.9%)	247 (1.0%)
	Native American	1 (1.6%)	1 (3.1%)	0.016	2 (3.1%)	0.016	203 (1.5%)	307 (1.3%)
	Other	7 (10.9%)	7 (15.6%)	0.031	10 (15.6%)	−0.047	508 (3.8%)	979 (4.0%)
Stable living	No	20 (31.7%)	19 (30.6%)	0.031	22 (36.7%)	0.063	2,189 (17.1%)	3,865 (16.5%)
	Yes	43 (68.3%)	43 (69.4%)	−0.031	38 (63.3%)	−0.078	10,631 (82.9%)	19,499 (83.5%)
Employment	Unemployed	33 (52.4%)	36 (58.1%	−0.031	36 (60.0%)	−0.031	5,989 (46.7%)	7,114 (30.4%)
	Employed full/part-time	30 (47.6%)	26 (41.9%	0.031	24 (40.0%)	0.016	6,831 (53.3%)	16,249 (69.6%)

^a^Transgender sample was matched to the female sample and independently matched to the male sample; ^b^Whole Cisgender Sample was not matched; ^c^SMD standardized mean difference.

**Table 2:** Comparison of primary substance used across gender group.

	**Matched Samples** ^**a**^			**Whole cisgender sample** ^**a**^	
**N (%)**	**Transgender (*****n** =* **64)**	**Female (*****n** =* **64)**	**Male (*****n** =* **64)**	**Female(*****n** =* **13,461)**	**Male (*****n** =* **24,512)**
Alcohol	21 (35.0%)	22 (35.5%)	23 (36.5%)	6,394 (49.3%)	10,397 (43.2%)
Marijuana	8 (13.3%)	6 (9.7%)	8 (12.7%)	552 (4.3%)	1,414 (5.9%)
Heroin	9 (15.0%)	4 (6.5%)	5 (7.9%)	1,817 (14.0%)	3,689 (15.3%)
Opiates	4 (6.7%)	6 (9.7%)	15 (23.8%)	1,424 (11.0%)	3,095 (12.9%)
Cocaine	2 (3.3%)	8 (12.9%)	1 (1.6%)	756 (5.8%)	1,763 (7.3%)
Methamphetamines	8 (13.3%)	6 (9.7%)	8 (12.7%)	808 (6.2%)	1,620 (6.7%)
Benzodiazepines	3 (5.0%)	2 (3.2%)	3 (4.8%)	559 (4.3%)	862 (3.6%)
Hallucinogens	0 (0.0%)	0 (0.0%)	0 (0.0%)	44 (0.3%)	132 (0.5%)
Amphetamines	0 (0.0%)	2 (3.2%)	0 (0.0%)	168 (1.3%)	330 (1.4%)
Club Drugs	1 (1.7%)	2 (3.2%)	0 (0.0%)	36 (0.3%)	94 (0.4%)
Synthetic drugs	1 (1.7%)	0 (0.0%)	0 (0.0%)	15 (0.1%)	53 (0.2%)
Stimulants	0 (0.0%)	0 (0.0%)	0 (0.0%)	20 (0.2%)	25 (0.1%)
Inhalants	0 (0.0%)	0 (0.0%)	0 (0.0%)	18 (0.1%)	40 (0.2%)
Other	3 (5.0%)	4 (6.5%)	0 (0.0%)	368 (2.8%)	527 (2.2%)

^a^Transgender sample was matched to the female sample and independently matched to the male sample; ^b^Whole Cisgender Sample was not matched.

As shown in Table 3, there were few statistically significant differences between the transgender sample and either of the cisgender samples on the intake variables; all non-significant comparisons also had small effect sizes (i.e., d > 0.5, ω > 0.3). The transgender sample was significantly higher than the cisgender male sample on PTSD symptoms (PCL-C scale), with a medium effect (*p* = 0.001, d = 0.57) but not significantly different than the cisgender female sample.

**Table 3:** Comparison between samples on mental health and SUD treatment outcome variables.

		**Matched cisgender samples**			
**Mental health and SUD variables**	**Transgender**	**Women**		**Men**	
	**(*****n** =* **64)**	**(*****n** =* **64)**		**(*****n** =* **64)**	
			
CAGE	2.90(1.32)	3.08 (1.30)	*P =* 0.50 d = 0.11	3.18 (1.21)	*p =* 0.11 d = 0.27
Patient health questionnaire−9	16.2 (7.01)	17.4 (6.75)	*p =* 0.63 d = 0.09	16.2 (6.96)	*p =* 0.90 d = 0.02
Generalized anxiety disorder−7	15.4 (5.14)	14.5 (5.54)	*p =* 0.08 d = 0.32	14.0 (6.02)	*p =* 0.09 d = 0.29
PRIME screening for psychosis	38.3(23.3)	28.6(17.9)	*p =* 0.06 d = 0.37	32.8(22.2)	*p =* 0.25 d = 0.18
Altman self-rating mania scale	9.89(4.65)	9.31(4.11)	*p =* 0.59 d = 0.09	9.74(4.87)	*p =* 0.83 d = 0.04
PTSD checklist–civilian version	17.3 (6.76)	16.2 (5.96)	*p =* 0.54 d = 0.10	**14.7(6.52)**	***p** **=*** **0.001 d** **=** **0.57**
Columbia suicide-severity rating scale screening version	1.81 (2.40)	**0.89 (1.38)**	***p** **<*** **0.001 d** **=** **1.15**	**0.98 (1.59)**	***p** **<*** **0.001 d** **=** **1.13**
DSM-5 symptoms of SUD	8.69 (3.59)	8.84 (3.17)	*p =* 0.31 d = 0.17	8.86 (3.34)	*p =* 0.63 d = 0.09
Number of substances use past 30 days	3.49(3.10)	3.45 (2.52)	*p =* 0.05 d = 0.37	3.48 (2.23)	*p =* 0.87 d = 0.03
Events before treatment	2.06 (1.52)	1.91 (1.05)	*p =* 0.71 d = 0.06	1.89 (1.18)	*p =* 0.11 d = 0.26
**SUD treatment outcome variables**	**(*****n** =* **49)**	**(*****n** =* **19)**	***p***<**0.00 1** ω= **0.45**	**(*****n** =* **28)**	***p***<**0.001** ω= **0.32**
Days in treatment	32.3 (22.2)	21.2 (32.4)	*p =* 0.15 d = 0.34	**19.5 (26.1)**	***p** **=*** **0.01 d** **=** **0.72**
Treatment completion	30 (61.2%)	10 (52.6%)	*p =* 0.71 ω= 0.05	12 (42.9%)	*p =* 0.19 ω= 0.15
Discharge AMA	7 (14.3%)	6 (31.6%)	*p =* 0.20 ω= 0.16	7 (25%)	*p =* 0.39 ω= 0.10

^*^Rounded from 0.047. The bold values indicate a statistically significant difference in the transgender sample.

One of the 64 members of the transgender sample met the criteria for anorexia nervosa on the eating disorder scale (EDDS), and one cisgender man met the criteria for binge eating disorder while none of the cisgender women met the criteria for any of the three eating disorders. A χ^2^ test, based on diagnosis, was not significant (χ^2^ = 2.01, df = 2, *p* = 0.36).

The transgender sample was significantly different from both cisgender samples for suicidality and with a large effect as measured by the CSSR scale. The transgender sample endorsed suicide as a reason for seeking treatment (*p* = 0.02) and screened positive for suicide risk significantly (*p* = 0.01) more frequently than matched cisgender men. Additionally, the transgender sample endorsed three of the six items on the CSSR scale significantly more often than either of the comparison samples, with small to medium effects (w = 0.20–0.032). They also endorsed an intent to carry out a suicide plan significantly more often than cisgender women (*p* = 0.03) but not cisgender men (see Table 4). Comparisons between transgender and cisgender participants were made for each item on all mental and behavioral health scales and are reported in the Supplementary material.

**Table 4:** Comparison between samples on intake suicidality.

		**Matched cisgender samples**			
**Suicide variables at intake**	**Transgender (*****n** =* **64)**	**Women (*****n** =* **64)**		**Men (*****n** =* **64)**	
Suicide as a reason for entering treatment	19 (39.7%)	**9** **(14.1%)**	***p** **=*** **0.05** **ω** **=** **0.19**	**7** **(10.9%)**	***p** **=*** **0.02** **ω** **=** **0.23**
Screening: Have you ever done anything started to do anything, or prepared to do anything to end your life?	25 (39.1%)	17 (26.6%)	*p =* 0.19 ω = 0.19	**11 (17.2%)**	***p** **=*** **0.01** **ω** **=** **0.24**
**Items of the Columbia suicide severity rating scale**					
1. In the month before treatment did you ever wish you were dead, or wish you could go to sleep and never wake up?	31 (48.4%)	29 (45.3%)	*p =* 0.86 ω = 0.03	29 (45.3%)	*p =* 0.86 ω = 0.03
2. In the month before treatment did you ever think about how you might kill yourself?	29 (45.3%)	**16** **(25%)**	***p** **=*** **0.03** **ω** **=** **0.21**	18 (28.1%)	*p =* 0.07 ω = 0.18
3. In the month before treatment did you ever think about how you might kill yourself?	24 (37.5%)	**7 (10.9%)**	***p*** **<** **0.01** **ω** **=** **0.31**	**11 (17.2%)**	***p** **=*** **0.02** **ω** **=** **0.23**
4. In the month before treatment did you ever have these thoughts and some intention of acting on them?	17 (26.6%)	**5 (7.8%)**	***p** **=*** **0.01** **ω** **=** **0.25**	**7** **(10.9%)**	***p** **=*** **0.04** **ω** **=** **0.20**
5. In the month before treatment did you start to work out the details of how to kill yourself?	12 (21.1%)	**2** **(3.2%)**	**p** **<** **0.01** **ω** **=** **0.28**	**3** **(5%)**	***p** **=*** **0.02** **ω** **=** **0.24**
6. Did you intend to carry out this plan?	10 (15.6%)	**2** **(3.1%)**	***p** **=*** **0.03** **ω** **=** **0.21**	3 (4.7%)	*p =* 0.08 ω = 0.18

Bold–Statistically significant difference at 0.05 as compared to the transgender sample.

There was some preliminary evidence that the transgender sample had more successful treatment outcomes than the cisgender samples. First, treatment attrition due to patient drop-out meant that fewer patients have treatment outcomes reported; however, a significantly higher proportion of the transgender sample had information recorded for outcome variables with medium effect when compared with cisgender women (*p* < 0.001, ω = 0.45) or cisgender men (*p* < 0.001, ω = 0.32). Second, this group remained in treatment longer (M = 32.3 days, SD = 22.2), on average, than cisgender men with a medium to large effect (M = 19.5, SD = 26.1, *p* = 0.03, d = 0.72).

## 4. Discussion

This study represents an initial step to understand the unique needs of gender minority populations as they seek treatment for SUDs. Previous research on SUD patterns among transgender individuals has been cross-sectional and does not include a demographically similar comparison sample. Our study addresses this gap by comparing those who self-identified as transgender with those who self-identified as cisgender male and female both on mental health and substance use variables collected at intake as well as outcome measures. There were several significant differences between the transgender and cisgender samples and the most important of which was the elevated prevalence of suicidal ideation, preparation, and action in the transgender sample.

The primary finding that the transgender sample had a higher suicide risk than the matched cisgender samples is consistent with the results of a 2021 systematic review, which found a preponderance of the evidence that members of transgender populations have an elevated risk of suicide that has often been associated with gender-related discrimination and stigma (53). Suicide risk has been found to be even higher among gender-minority individuals with a concurrent diagnosis of SUD (53). The findings of the current study are also consistent with previous cross-sectional studies which found that drug use patterns among transgender individuals in treatment are broadly comparable with those of cisgender individuals in substance use treatment (54, 55).

The transgender sample had the highest score on the PTSD symptoms scale of the three groups and was significantly higher than the sample of cisgender men. This is in line with previous research which found that discrimination experiences disproportionally affect transgender persons and increase the risk of PTSD (56). Exposure to traumatic stress has also been found to partially mediate the association between minority stress—due to gender status—and risk of suicide (57), which may partially explain the elevated risk of suicide found in the transgender sample.

This is the first study to compare treatment outcomes between a transgender sample and cisgender samples, though this study was limited to individuals who identified broadly as transgender. The results were indicative that transgender individuals in substance use treatment remained in treatment as long as cisgender women and longer than cisgender men. This may be due to the prevalence of suicidal ideation and actions among the transgender sample which has previously been associated with increased substance use treatment completion, possibly because disclosure of suicidal ideation by those in treatment to providers may indicate greater treatment engagement (58). Additionally, the transgender sample had more chronic health problems than cisgender women and had higher PTSD symptoms than cisgender men. These findings should be confirmed by future research and in turn inform treatment providers to develop culturally appropriate interventions for people with SUD who are transgender.

Limitations of this study include a somewhat small sample size and therefore requires further verification in larger samples. Additionally, limited survey options for gender identity (male, female, and transgender), implies several limitations: only those who were willing to identify as transgender for the purposes of the intake survey would be included, excluding those who identify as non-binary or other gender appellation that does not conform to this categorization and those who did not feel comfortable identifying as transgender in the context of SUD treatment. While fitting the above definition of transgender, it also excludes those who do not consider themselves to be within that category either because they simply identify as a man or woman without qualification or because the question may have been interpreted as asking for sex assigned at birth. An additional limitation of the gender options provided is that the only available term to indicate that an individual is transgender does not differentiate transgender sub-groups (i.e., transgender men or women and non-binary individuals) which are different populations. Future studies should include multifaceted gender identity questions to better contextualize the risks and outcomes of the gender minority populations.

While it was not possible for this study to investigate the different risks facing trans feminine, trans masculine, non-binary, and other gender non-conforming individuals, other studies have shown important differences in mental health disorder prevalence and sexual health behaviors when comparing these gender identities. For instance, a 2023 global sample of transgender and non-binary people found differences in substance use behaviors during the COVID-19 pandemic between transmasculine, transfeminine, and non-binary participants (59). Glick et al. (6) outline the various issues with different data collection methods that aim to understand the different gender minority populations. Importantly, all of the current procedures—including asking for sex at birth and gender identity as recommended by the CDC (5)—were not developed by individuals who were gender minorities themselves. Without being able to collect this information, researchers are not able to identify differences between sub-populations, and consequently, treatment providers will be unable to tailor treatments to the needs of these communities. While it is critical to design treatment interventions that address the needs of gender minority communities from the bottom up, the lack of information about differences between gender sub-communities inhibits treatment providers from adapting existing treatment protocols appropriately. It is imperative that the field of SUD treatment collects this information in a culturally competent way, to support SUD recovery for transgender persons.

## 5. Conclusion

This study shows that there are significant differences in the motivation for seeking SUD treatment between transgender and cisgender individuals and that transgender individuals may stay in treatment longer, on average, than cisgender individuals. The heightened levels of suicidal thoughts and actions in this population highlight the need for specialized mental healthcare that accounts for diverse gender identities and additional studies that assess if current SUD treatment is meeting the needs of transgender individuals.

## Data availability statement

The datasets presented in this article are not readily available because the data were collected by a private firm and shared only with one research group at the Department of Psychiatry of Johns Hopkins University School of Medicine. Summary data is available in the Supplementary material. Requests to access the datasets should be directed to mhochhe1@jh.edu.

## Ethics statement

The Johns Hopkins University Institutional Review Board reviewed and acknowledged that this study did not constitute human subjects research. Written informed consent to participate was not required in accordance with institutional and national guidelines.

## Author contributions

MH conceived study, determined method, preformed analysis, and wrote draft of manuscript. AH aided in study conception and determination of method, wrote sections, and edited the manuscript. JG and HG-D added sections and edited the manuscript and needed expertise to the study. All authors contributed to the article and approved the submitted version.
