# Supplementary material for: Transgender individuals are at higher risk for suicidal ideation and preparation than cisgender individuals in substance use treatment
Source: Front Psychiatry. 2023 Sep 13;14:1225673. doi: 10.3389/fpsyt.2023.1225673 (PMC10535091; doi:10.3389/fpsyt.2023.1225673)
Supplement: Supplementary file 1 [file Table_1.DOCX]

| **Table S1. Reason Endorsed for Entering Treatment** | | | |
| --- | --- | --- | --- |
|  | **Transgender**  **(n=64)** | **Matched Female**  **(n=64)** | **Matched Male**  **(n=64)** |
| Alcohol Abuse | 34 (53.1%) | 33 (51.6%) | 32 (50.0%) |
| Drug Abuse | 39 (60.9%) | 42 (65.6%) | 45 (70.3%) |
| Depression | 33 (51.6%) | 36 (56.2%) | 32 (50.0%) |
| Anxiety | 35 (54.7%) | 38 (59.4%) | 37 (57.8%) |
| Suicide | 19 (29.7%) | 9 (14.1%) | 7 (10.9%) |
| Eating Disorder | 6 (9.4%) | 7 (10.9%) | 2 3.1%) |
| Bipolar Disorder | 6 (9.4%) | 5 (7.8%) | 4 (6.2%) |

| **Table S2. Importance of successfully completing treatment** | | | |
| --- | --- | --- | --- |
|  | **Transgender,**  **N = 64^1^** | **Matched Female,**  **N = 64^1^** | **Matched Male,**  **N = 64^1^** |
| How important do you consider treating your alcohol or drug abuse to be? | | | |
| Not important | 1 (1.9%) | 0 (0%) | 0 (0%) |
| Somewhat important | 4 (7.4%) | 5 (8.5%) | 2 (3.4%) |
| Important | 6 (11%) | 11 (19%) | 5 (8.6%) |
| Very important | 43 (80%) | 43 (73%) | 51 (88%) |
| Unknown | 10 | 5 | 6 |
| How important do you consider treating your emotional health to be? | | | |
| Not important | 3 (6.5%) | 0 (0%) | 0 (0%) |
| Somewhat important | 1 (2.2%) | 2 (4.2%) | 2 (4.8%) |
| Important | 5 (11%) | 5 (10%) | 6 (14%) |
| Very important | 37 (80%) | 41 (85%) | 34 (81%) |
| Unknown | 18 | 16 | 22 |
| How important do you consider treating your eating disorder to be? | | | |
| Not important | 0 (0%) | 1 (14%) | 0 (0%) |
| Somewhat important | 4 (67%) | 1 (14%) | 0 (0%) |
| Important | 0 (0%) | 2 (29%) | 0 (0%) |
| Very important | 2 (33%) | 3 (43%) | 2 (100%) |
| Unknown | 58 | 57 | 62 |
| How important do you consider treating your other condition to be? | | | |
| Not important | 1 (14%) | 0 (0%) | 0 (0%) |
| Somewhat important | 0 (0%) | 0 (0%) | 1 (20%) |
| Important | 1 (14%) | 2 (33%) | 0 (0%) |
| Very important | 5 (71%) | 4 (67%) | 4 (80%) |
| Unknown | 57 | 58 | 59 |
| ^1^ n (%) | | | |

| **Table S3. Intake screening questions** | | | | |
| --- | --- | --- | --- | --- |
|  | **Transgender, N = 64^1^** | **Matched Female, N = 64^1^** | **Matched Male, N = 64^1^** |  |
| Depression Screening: Did you often feel down, depressed, or hopeless? | | | | |
| No | 14 (22%) | 12 (19%) | 8 (13%) |  |
| Yes | 49 (78%) | 50 (81%) | 52 (87%) |  |
| Unknown | 1 | 2 | 4 |  |
| Depression Screening: Did you often feel little interest or pleasure in doing things? | | | | |
| No | 13 (21%) | 17 (27%) | 9 (15%) |  |
| Yes | 50 (79%) | 45 (73%) | 51 (85%) |  |
| Unknown | 1 | 2 | 4 |  |
| Anxiety Screening: Do you often feel nervous, anxious, or on edge? | | | | |
| No | 7 (11%) | 7 (11%) | 5 (8.5%) |  |
| Yes | 56 (89%) | 55 (89%) | 54 (92%) |  |
| Unknown | 1 | 2 | 5 |  |
| Anxiety Screening: Have you been unable to stop or control worrying? | | | | |
| No | 18 (29%) | 21 (34%) | 17 (29%) |  |
| Yes | 45 (71%) | 40 (66%) | 42 (71%) |  |
| Unknown | 1 | 3 | 5 |  |
| PTSD Screenings: Did you often have repeated, disturbing memories, thoughts, or images of a stressful experience from the past? | | | | |
| No | 20 (32%) | 15 (24%) | 17 (28%) |  |
| Yes | 43 (68%) | 47 (76%) | 43 (72%) |  |
| Unknown | 1 | 2 | 4 |  |
| PTSD Screenings: Did you often feel very upset when something reminded you of a stressful experience from the past? | | | | |
| No | 19 (30%) | 15 (24%) | 8 (13%) |  |
| Yes | 44 (70%) | 47 (76%) | 52 (87%) |  |
| Unknown | 1 | 2 | 4 |  |
|  |  |  |  |  |
| Mania Screenings: Were there any periods when you were unusually active, talkative, or irritable for a day or more at a time? | | | | |
| No | 6 (30%) | 7 (32%) | 11 (34%) |  |
| Yes | 14 (70%) | 15 (68%) | 21 (66%) |  |
| Unknown | 44 | 42 | 32 |  |
| Psychotic Screening: Did you ever see things or hear voices that others couldn’t see or hear? | | | | |
| No | 12 (60%) | 17 (77%) | 27 (84%) |  |
| Yes | 8 (40%) | 5 (23%) | 5 (16%) |  |
| Unknown | 44 | 42 | 32 |  |
| Psychotic Screening: Did you ever feel that people were paying special attention to you or were out to get you? | | | | |
| No | 10 (50%) | 15 (68%) | 18 (56%) |  |
| Yes | 10 (50%) | 7 (32%) | 14 (44%) |  |
| Unknown | 44 | 42 | 32 |  |
| Eating Disorder Screenings: Did you ever worry that you had lost control over how much you were eating? | | | | |
| No | 41 (68%) | 38 (63%) | 44 (76%) |  |
| Yes | 19 (32%) | 22 (37%) | 14 (24%) |  |
| Unknown | 4 | 4 | 6 |  |
| Eating Disorder Screenings: Did you make yourself vomit? | | | | |
| No | 49 (82%) | 52 (87%) | 53 (91%) |  |
| Yes | 11 (18%) | 8 (13%) | 5 (8.6%) |  |
| Unknown | 4 | 4 | 6 |  |
| Eating Disorder Screenings: Did you every believe yourself to be fat when others said you were too thin? | | | | |
| No | 47 (78%) | 38 (63%) | 52 (90%) |  |
| Yes | 13 (22%) | 22 (37%) | 6 (10%) |  |
| Unknown | 4 | 4 | 6 |  |
| Eating Disorder Screenings: Have you lost more than 14 pounds in a three-month period in the last few years? | | | | |
| No | 31 (52%) | 30 (50%) | 24 (41%) |  |
| Yes | 29 (48%) | 30 (50%) | 34 (59%) |  |
| Unknown | 4 | 4 | 6 |  |
| Eating Disorder Screenings Did you ever feel that food dominated your life? | | | | |
| No | 51 (85%) | 49 (82%) | 50 (86%) |  |
| Yes | 9 (15%) | 11 (18%) | 8 (14%) |  |
| Unknown | 4 | 4 | 6 |  |
| n (%) |  |  |  |  |

| **Table S4. CAGE Screening (adjusted for each drug of choice)** | | | |
| --- | --- | --- | --- |
|  | **Transgender,**  **N = 64^1^** | **Matched Female,**  **N = 64^1^** | **Matched Male,**  **N = 64^1^** |
| Intake Screenings: Did you ever feel you ought to cut down on your drinking or drug use? | | | |
| No | 15 (25%) | 11 (21%) | 9 (18%) |
| Yes | 44 (75%) | 42 (79%) | 42 (82%) |
| Unknown | 5 | 11 | 13 |
| Intake Screenings: Did you ever get annoyed by people who criticized your drinking or drug use? | | | |
| No | 17 (29%) | 16 (30%) | 15 (29%) |
| Yes | 42 (71%) | 37 (70%) | 36 (71%) |
| Unknown | 5 | 11 | 13 |
| Intake Screenings: Did you ever feel bad or guilty about your drinking or drug use? | | | |
| No | 17 (29%) | 11 (21%) | 7 (14%) |
| Yes | 42 (71%) | 42 (79%) | 44 (86%) |
| Unknown | 5 | 11 | 13 |
| Intake Screenings: Did you ever have a drink or use drugs first thing in the morning to steady your nerves or to get rid of a hangover? | | | |
| No | 16 (27%) | 11 (21%) | 11 (22%) |
| Yes | 43 (73%) | 42 (79%) | 40 (78%) |
| Unknown | 5 | 11 | 13 |
| ^1^ n (%) | | | |

| **Table S5. Events Before Treatment** | | | |
| --- | --- | --- | --- |
|  | **Transgender, N = 64^1^** | **Matched Female, N = 64^1^** | **Matched Male, N = 64^1^** |
| Family and/or friends asked me or told me to go to treatment | | | |
| No | 34 (53%) | 27 (42%) | 25 (39%) |
| Yes | 30 (47%) | 37 (58%) | 39 (61%) |
| I became scared or upset by the way I was feeling | | | |
| No | 60 (94%) | 59 (92%) | 63 (98%) |
| Yes | 4 (6.2%) | 5 (7.8%) | 1 (1.6%) |
| I became tired of living this way | | | |
| No | 20 (31%) | 18 (28%) | 17 (27%) |
| Yes | 44 (69%) | 46 (72%) | 47 (73%) |
| I was arrested and/or jailed | | | |
| No | 56 (88%) | 61 (95%) | 55 (86%) |
| Yes | 8 (12%) | 3 (4.7%) | 9 (14%) |
| I was caught driving under the influence | | | |
| No | 59 (92%) | 64 (100%) | 63 (98%) |
| Yes | 5 (7.8%) | 0 (0%) | 1 (1.6%) |
| I was hospitalized | | | |
| No | 45 (70%) | 52 (81%) | 54 (84%) |
| Yes | 19 (30%) | 12 (19%) | 10 (16%) |
| I overdosed | | | |
| No | 54 (84%) | 59 (92%) | 59 (92%) |
| Yes | 10 (16%) | 5 (7.8%) | 5 (7.8%) |
| I got alcohol poisoning | | | |
| No | 62 (97%) | 61 (95%) | 63 (98%) |
| Yes | 2 (3.1%) | 3 (4.7%) | 1 (1.6%) |
| I was ordered to go to treatment by a court or the criminal justice system | | | |
| No | 62 (97%) | 63 (98%) | 63 (98%) |
| Yes | 2 (3.1%) | 1 (1.6%) | 1 (1.6%) |
| I was involuntarily committed | | | |
| No | 63 (98%) | 63 (98%) | 64 (100%) |
| Yes | 1 (1.6%) | 1 (1.6%) | 0 (0%) |
| ^1^ n (%) | | | |

| **Table S6. Life Goals After Treatment** | | | |
| --- | --- | --- | --- |
|  | **Transgender,**  **N = 64^1^** | **Matched Female,**  **N = 64^1^** | **Matched Male,**  **N = 64^1^** |
| Stay alive | | | |
| Not specified as a goal | 14 (67%) | 26 (44%) | 30 (50%) |
| Specified as a goal | 7 (33%) | 33 (56%) | 30 (50%) |
| Unknown/Missing | 43 | 5 | 4 |
| Regain the trust of my family | | | |
| Not specified as a goal | 15 (71%) | 20 (34%) | 18 (30%) |
| Specified as a goal | 6 (29%) | 39 (66%) | 42 (70%) |
| Unknown/Missing | 43 | 5 | 4 |
| Return home | | | |
| Not specified as a goal | 16 (76%) | 39 (66%) | 46 (77%) |
| Specified as a goal | 5 (24%) | 20 (34%) | 14 (23%) |
| Unknown/Missing | 43 | 5 | 4 |
| Get my own place to live | | | |
| Not specified as a goal | 15 (71%) | 39 (66%) | 40 (67%) |
| Specified as a goal | 6 (29%) | 20 (34%) | 20 (33%) |
| Unknown/Missing | 43 | 5 | 4 |
| Stay out of jail or prison | | | |
| Not specified as a goal | 17 (81%) | 48 (81%) | 38 (63%) |
| Specified as a goal | 4 (19%) | 11 (19%) | 22 (37%) |
| Unknown/Missing | 43 | 5 | 4 |
| Get a car/regain my drivers license | | | |
| Not specified as a goal | 16 (76%) | 47 (80%) | 49 (82%) |
| Specified as a goal | 5 (24%) | 12 (20%) | 11 (18%) |
| Unknown/Missing | 43 | 5 | 4 |
| Return to school | | | |
| Not specified as a goal | 15 (71%) | 42 (71%) | 50 (83%) |
| Specified as a goal | 6 (29%) | 17 (29%) | 10 (17%) |
| Unknown/Missing | 43 | 5 | 4 |
| Regain my job, or get a similar one | | | |
| Not specified as a goal | 16 (76%) | 43 (73%) | 47 (78%) |
| Specified as a goal | 5 (24%) | 16 (27%) | 13 (22%) |
| Unknown/Missing | 43 | 5 | 4 |
| Regain custody of my children | | | |
| Not specified as a goal | 20 (95%) | 50 (85%) | 56 (93%) |
| Specified as a goal | 1 (4.8%) | 9 (15%) | 4 (6.7%) |
| Unknown/Missing | 43 | 5 | 4 |
| Get back together with my partner or spouse | | | |
| Not specified as a goal | 20 (95%) | 49 (83%) | 52 (87%) |
| Specified as a goal | 1 (4.8%) | 10 (17%) | 8 (13%) |
| Unknown/Missing | 43 | 5 | 4 |
| None of these are relevant life goals for me | | | |
| Not specified as a goal | 20 (95%) | 59 (100%) | 57 (95%) |
| Specified as a goal | 1 (4.8%) | 0 (0%) | 3 (5.0%) |
| Unknown/Missing | 43 | 5 | 4 |
| Other | | | |
| Not specified as a goal | 15 (71%) | 56 (95%) | 52 (87%) |
| Specified as a goal | 6 (29%) | 3 (5.1%) | 8 (13%) |
| Unknown/Missing | 43 | 5 | 4 |
| ^1^ n (%) | | | |

| **Table S7. Substance Use Prior to Treatment** | | | |
| --- | --- | --- | --- |
|  | **Transgender,**  **N = 64^1^** | **Matched Female,**  **N = 64^1^** | **Matched Male,**  **N = 64^1^** |
| Alcohol usage in 30 days before treatment | | | |
| Have never used | 3 (4.9%) | 4 (6.5%) | 6 (9.4%) |
| Have used, but not in the 30 days before treatment | 15 (25%) | 15 (24%) | 12 (19%) |
| Used in the 30 days before treatment, but not heavily | 18 (30%) | 15 (24%) | 22 (34%) |
| Used heavily in the 30 days before treatment | 25 (41%) | 28 (45%) | 24 (38%) |
| Unknown/Missing | 3 | 2 | 0 |
| Marijuana usage in 30 days before treatment | | | |
| Have never used | 9 (15%) | 9 (15%) | 5 (7.8%) |
| Have used, but not in the 30 days before treatment | 17 (28%) | 19 (31%) | 22 (34%) |
| Used in the 30 days before treatment, but not heavily | 12 (20%) | 12 (19%) | 17 (27%) |
| Used heavily in the 30 days before treatment | 23 (38%) | 22 (35%) | 20 (31%) |
| Unknown/Missing | 3 | 2 | 0 |
| Amphetamine usage in 30 days before treatment | | | |
| Have never used | 22 (36%) | 22 (35%) | 29 (45%) |
| Have used, but not in the 30 days before treatment | 27 (44%) | 21 (34%) | 21 (33%) |
| Used in the 30 days before treatment, but not heavily | 9 (15%) | 9 (15%) | 12 (19%) |
| Used heavily in the 30 days before treatment | 3 (4.9%) | 10 (16%) | 2 (3.1%) |
| Unknown/Missing | 3 | 2 | 0 |
| Cocaine usage in 30 days before treatment | | | |
| Have never used | 21 (34%) | 17 (27%) | 18 (28%) |
| Have used, but not in the 30 days before treatment | 22 (36%) | 29 (47%) | 28 (44%) |
| Used in the 30 days before treatment, but not heavily | 10 (16%) | 8 (13%) | 12 (19%) |
| Used heavily in the 30 days before treatment | 8 (13%) | 8 (13%) | 6 (9.4%) |
| Unknown/Missing | 3 | 2 | 0 |
| Methamphetamine usage in 30 days before treatment | | | |
| Have never used | 30 (49%) | 31 (50%) | 35 (55%) |
| Have used, but not in the 30 days before treatment | 14 (23%) | 13 (21%) | 10 (16%) |
| Used in the 30 days before treatment, but not heavily | 7 (11%) | 6 (9.7%) | 9 (14%) |
| Used heavily in the 30 days before treatment | 10 (16%) | 12 (19%) | 10 (16%) |
| Unknown/Missing | 3 | 2 | 0 |
| Hallucinogen usage in 30 days before treatment | | | |
| Have never used | 25 (41%) | 30 (48%) | 20 (31%) |
| Have used, but not in the 30 days before treatment | 27 (44%) | 18 (29%) | 35 (55%) |
| Used in the 30 days before treatment, but not heavily | 4 (6.6%) | 12 (19%) | 7 (11%) |
| Used heavily in the 30 days before treatment | 5 (8.2%) | 2 (3.2%) | 2 (3.1%) |
| Unknown/Missing | 3 | 2 | 0 |
| Opiate usage in 30 days before treatment | | | |
| Have never used | 23 (38%) | 25 (40%) | 15 (23%) |
| Have used, but not in the 30 days before treatment | 21 (34%) | 24 (39%) | 21 (33%) |
| Used in the 30 days before treatment, but not heavily | 6 (9.8%) | 4 (6.5%) | 10 (16%) |
| Used heavily in the 30 days before treatment | 11 (18%) | 9 (15%) | 18 (28%) |
| Unknown/Missing | 3 | 2 | 0 |
| Benzodiazepine usage in 30 days before treatment | | | |
| Have never used | 18 (30%) | 24 (39%) | 24 (38%) |
| Have used, but not in the 30 days before treatment | 24 (39%) | 12 (19%) | 16 (25%) |
| Used in the 30 days before treatment, but not heavily | 6 (9.8%) | 14 (23%) | 19 (30%) |
| Used heavily in the 30 days before treatment | 13 (21%) | 12 (19%) | 5 (7.8%) |
| Unknown/Missing | 3 | 2 | 0 |
| Stimulant usage in 30 days before treatment | | | |
| Have never used | 37 (61%) | 43 (69%) | 48 (75%) |
| Have used, but not in the 30 days before treatment | 20 (33%) | 16 (26%) | 12 (19%) |
| Used in the 30 days before treatment, but not heavily | 2 (3.3%) | 3 (4.8%) | 3 (4.7%) |
| Used heavily in the 30 days before treatment | 2 (3.3%) | 0 (0%) | 1 (1.6%) |
| Unknown/Missing | 3 | 2 | 0 |
| Heroin usage in 30 days before treatment | | | |
| Have never used | 37 (61%) | 40 (65%) | 37 (58%) |
| Have used, but not in the 30 days before treatment | 10 (16%) | 11 (18%) | 12 (19%) |
| Used in the 30 days before treatment, but not heavily | 1 (1.6%) | 6 (9.7%) | 4 (6.2%) |
| Used heavily in the 30 days before treatment | 13 (21%) | 5 (8.1%) | 11 (17%) |
| Unknown/Missing | 3 | 2 | 0 |
| Inhalant usage in 30 days before treatment | | | |
| Have never used | 40 (66%) | 47 (76%) | 53 (83%) |
| Have used, but not in the 30 days before treatment | 17 (28%) | 12 (19%) | 10 (16%) |
| Used in the 30 days before treatment, but not heavily | 2 (3.3%) | 1 (1.6%) | 0 (0%) |
| Used heavily in the 30 days before treatment | 2 (3.3%) | 2 (3.2%) | 1 (1.6%) |
| Unknown/Missing | 3 | 2 | 0 |
| Club drug usage in 30 days before treatment | | | |
| Have never used | 28 (46%) | 28 (45%) | 24 (38%) |
| Have used, but not in the 30 days before treatment | 24 (39%) | 25 (40%) | 34 (53%) |
| Used in the 30 days before treatment, but not heavily | 4 (6.6%) | 3 (4.8%) | 4 (6.2%) |
| Used heavily in the 30 days before treatment | 5 (8.2%) | 6 (9.7%) | 2 (3.1%) |
| Unknown/Missing | 3 | 2 | 0 |
| Synthetic drug usage in 30 days before treatment | | | |
| Have never used | 38 (62%) | 46 (74%) | 53 (83%) |
| Have used, but not in the 30 days before treatment | 18 (30%) | 14 (23%) | 10 (16%) |
| Used in the 30 days before treatment, but not heavily | 3 (4.9%) | 1 (1.6%) | 0 (0%) |
| Used heavily in the 30 days before treatment | 2 (3.3%) | 1 (1.6%) | 1 (1.6%) |
| Unknown/Missing | 3 | 2 | 0 |
| Other drug usage in 30 days before treatment | | | |
| Have never used | 47 (77%) | 49 (79%) | 55 (86%) |
| Have used, but not in the 30 days before treatment | 7 (11%) | 10 (16%) | 8 (12%) |
| Used in the 30 days before treatment, but not heavily | 2 (3.3%) | 1 (1.6%) | 1 (1.6%) |
| Used heavily in the 30 days before treatment | 5 (8.2%) | 2 (3.2%) | 0 (0%) |
| Unknown/Missing | 3 | 2 | 0 |
| ^1^ n (%) | | | |

| **Table S8. Substance Use Symptoms** | | | |
| --- | --- | --- | --- |
|  | **Transgender,**  **N = 64^1^** | **Matched Female,**  **N = 64^1^** | **Matched Male,**  **N = 64^1^** |
| Intake: In the year before entering treatment, did you have times when you ended up drinking more or taking more drugs than you intended? Or drinking or drugging longer than you intended? | | | |
| No | 11 (18%) | 10 (16%) | 6 (9.4%) |
| Yes | 50 (82%) | 53 (84%) | 58 (91%) |
| Unknown/Missing | 3 | 1 | 0 |
| Intake: In the year before entering treatment, did you more than once want to cut down or stop drinking or drugging, or tried to, but couldn’t. | | | |
| No | 13 (21%) | 13 (21%) | 10 (16%) |
| Yes | 48 (79%) | 50 (79%) | 54 (84%) |
| Unknown/Missing | 3 | 1 | 0 |
| Intake: In the year before entering treatment, did you spend a lot of time drinking or doing drugs? Or being sick or getting over other aftereffects? | | | |
| No | 12 (20%) | 10 (16%) | 12 (19%) |
| Yes | 49 (80%) | 53 (84%) | 52 (81%) |
| Unknown/Missing | 3 | 1 | 0 |
| Intake: In the year before entering treatment, did you want a drink or drugs so badly you couldn’t think of anything else? | | | |
| No | 17 (28%) | 19 (30%) | 18 (28%) |
| Yes | 44 (72%) | 44 (70%) | 46 (72%) |
| Unknown/Missing | 3 | 1 | 0 |
| Intake: In the year before entering treatment, did you find that drinking or using drugs – or being sick from doing so – often interfered with taking care of your home, school, family, or friends? | | | |
| No | 13 (21%) | 14 (22%) | 16 (25%) |
| Yes | 48 (79%) | 49 (78%) | 48 (75%) |
| Unknown/Missing | 3 | 1 | 0 |
| Intake: In the year before entering treatment, did you continue drink or drug even though it was causing trouble with your family or friends? | | | |
| No | 10 (16%) | 7 (11%) | 9 (14%) |
| Yes | 51 (84%) | 56 (89%) | 55 (86%) |
| Unknown/Missing | 3 | 1 | 0 |
| Intake: In the year before entering treatment, did you give up or cut back on activities that were important or interesting to you, or gave you pleasure, in order to drink or drug? | | | |
| No | 16 (26%) | 13 (21%) | 13 (20%) |
| Yes | 45 (74%) | 50 (79%) | 51 (80%) |
| Unknown/Missing | 3 | 1 | 0 |
| Intake: In the year before entering treatment, did you more than once get into situations while or after drinking or drugging that increased your chances of getting hurt | | | |
| No | 14 (23%) | 16 (25%) | 15 (23%) |
| Yes | 47 (77%) | 47 (75%) | 49 (77%) |
| Unknown/Missing | 3 | 1 | 0 |
| Intake: In the year before entering treatment, did you continue to drink or drug even though it was making you feel depressed or anxious or adding to another health problem? Or after having had a memory blackout? | | | |
| No | 10 (16%) | 12 (19%) | 10 (16%) |
| Yes | 51 (84%) | 51 (81%) | 54 (84%) |
| Unknown/Missing | 3 | 1 | 0 |
| Tolerance in year before entering treatment | | | |
| No | 8 (13%) | 11 (17%) | 11 (17%) |
| Yes | 53 (87%) | 52 (83%) | 53 (83%) |
| Unknown/Missing | 3 | 1 | 0 |
| Withdrawal in year before treatment | | | |
| No | 17 (28%) | 11 (17%) | 17 (27%) |
| Yes | 44 (72%) | 52 (83%) | 47 (73%) |
| Unknown/Missing | 3 | 1 | 0 |
| ^1^ n (%) | | | |

| **Table S9. Physical Health** | | | |
| --- | --- | --- | --- |
|  | **Transgender, N = 64^1^** | **Matched Female, N = 64^1^** | **Matched Male, N = 64^1^** |
| Has asthma | | | |
| No | 51 (81%) | 59 (95%) | 51 (86%) |
| Yes | 12 (19%) | 3 (4.8%) | 8 (14%) |
| Unknown/Missing | 1 | 2 | 5 |
| Has cancer | | | |
| No | 63 (100%) | 62 (100%) | 59 (100%) |
| Yes | 0 (0%) | 0 (0%) | 0 (0%) |
| Unknown/Missing | 1 | 2 | 5 |
| Has COPD/emphysema | | | |
| No | 62 (98%) | 60 (97%) | 59 (100%) |
| Yes | 1 (1.6%) | 2 (3.2%) | 0 (0%) |
| Unknown/Missing | 1 | 2 | 5 |
| Has coronary heart disease/heart attach | | | |
| No | 63 (100%) | 61 (98%) | 59 (100%) |
| Yes | 0 (0%) | 1 (1.6%) | 0 (0%) |
| Unknown/Missing | 1 | 2 | 5 |
| Has diabetes | | | |
| No | 60 (95%) | 62 (100%) | 57 (97%) |
| Yes | 3 (4.8%) | 0 (0%) | 2 (3.4%) |
| Unknown/Missing | 1 | 2 | 5 |
| Has HepC | | | |
| No | 63 (100%) | 57 (92%) | 56 (95%) |
| Yes | 0 (0%) | 5 (8.1%) | 3 (5.1%) |
| Unknown/Missing | 1 | 2 | 5 |
| Has HIV/AIDS | | | |
| No | 61 (97%) | 62 (100%) | 59 (100%) |
| Yes | 2 (3.2%) | 0 (0%) | 0 (0%) |
| Unknown/Missing | 1 | 2 | 5 |
| Has hypertension | | | |
| No | 58 (92%) | 58 (94%) | 56 (95%) |
| Yes | 5 (7.9%) | 4 (6.5%) | 3 (5.1%) |
| Unknown/Missing | 1 | 2 | 5 |
| Has organ transplantation | | | |
| No | 63 (100%) | 62 (100%) | 59 (100%) |
| Yes | 1 | 2 | 5 |
| Has renal/kidney failure | | | |
| No | 63 (100%) | 62 (100%) | 59 (100%) |
| Yes | 1 | 2 | 5 |
| Has sickle cell anemia | | | |
| No | 63 (100%) | 62 (100%) | 59 (100%) |
| Yes | 0 (0%) | 0 (0%) | 0 (0%) |
| Unknown/Missing | 1 | 2 | 5 |
| Had a stroke | | | |
| No | 63 (100%) | 62 (100%) | 59 (100%) |
| Yes | 0 (0%) | 0 (0%) | 0 (0%) |
| Unknown/Missing | 1 | 2 | 5 |
| Has other chronic health condition | | | |
| No | 56 (89%) | 56 (90%) | 56 (95%) |
| Yes | 7 (11%) | 6 (9.7%) | 3 (5.1%) |
| Unknown/Missing | 1 | 2 | 5 |
| ^1^ n (%) | | | |

| **Table S10. PHQ –9** | | | |
| --- | --- | --- | --- |
|  | **Transgender,**  **N = 64^1^** | **Matched Female,**  **N = 64^1^** | **Matched Male,**  **N = 64^1^** |
| Little interest or pleasure | | | |
| Not at all | 6 (11%) | 3 (5.9%) | 4 (7.1%) |
| Several days | 17 (31%) | 12 (24%) | 9 (16%) |
| More than half the days | 16 (29%) | 14 (27%) | 17 (30%) |
| Nearly every day | 16 (29%) | 22 (43%) | 26 (46%) |
| Unknown/Missing/Screened negative | 9 | 13 | 8 |
| Feeling down | | | |
| Not at all | 2 (3.6%) | 0 (0%) | 4 (7.1%) |
| Several days | 12 (22%) | 13 (25%) | 12 (21%) |
| More than half the days | 16 (29%) | 10 (20%) | 15 (27%) |
| Nearly every day | 25 (45%) | 28 (55%) | 25 (45%) |
| Unknown/Missing/Screened negative | 9 | 13 | 8 |
| Trouble falling or staying asleep | | | |
| Not at all | 10 (18%) | 6 (12%) | 3 (5.4%) |
| Several days | 10 (18%) | 4 (7.8%) | 14 (25%) |
| More than half the days | 12 (22%) | 11 (22%) | 11 (20%) |
| Nearly every day | 23 (42%) | 30 (59%) | 28 (50%) |
| Unknown/Missing/Screened negative | 9 | 13 | 8 |
| Feeling tired | | | |
| Not at all | 6 (11%) | 6 (12%) | 7 (12%) |
| Several days | 12 (22%) | 5 (9.8%) | 11 (20%) |
| More than half the days | 15 (27%) | 11 (22%) | 16 (29%) |
| Nearly every day | 22 (40%) | 29 (57%) | 22 (39%) |
| Unknown/Missing/Screened negative | 9 | 13 | 8 |
| Poor appetite | | | |
| Not at all | 13 (24%) | 9 (18%) | 13 (23%) |
| Several days | 6 (11%) | 10 (20%) | 12 (21%) |
| More than half the days | 12 (22%) | 7 (14%) | 13 (23%) |
| Nearly every day | 24 (44%) | 25 (49%) | 18 (32%) |
| Unknown/Missing/Screened negative | 9 | 13 | 8 |
| Feeling bad | | | |
| Not at all | 4 (7.3%) | 3 (5.9%) | 6 (11%) |
| Several days | 7 (13%) | 8 (16%) | 7 (12%) |
| More than half the days | 16 (29%) | 9 (18%) | 12 (21%) |
| Nearly every day | 28 (51%) | 31 (61%) | 31 (55%) |
| Unknown/Missing/Screened negative | 9 | 13 | 8 |
| Trouble concentrating | | | |
| Not at all | 9 (16%) | 10 (20%) | 8 (14%) |
| Several days | 13 (24%) | 5 (9.8%) | 14 (25%) |
| More than half the days | 12 (22%) | 13 (25%) | 17 (30%) |
| Nearly every day | 21 (38%) | 23 (45%) | 17 (30%) |
| Unknown/Missing/Screened negative | 9 | 13 | 8 |
| Moving or speaking slowly | | | |
| Not at all | 24 (44%) | 19 (37%) | 21 (38%) |
| Several days | 10 (18%) | 8 (16%) | 14 (25%) |
| More than half the days | 10 (18%) | 9 (18%) | 9 (16%) |
| Nearly every day | 11 (20%) | 15 (29%) | 12 (21%) |
| Unknown/Missing/Screened negative | 9 | 13 | 8 |
| Thoughts that you would be better off dead | | | |
| Not at all | 19 (35%) | 25 (49%) | 24 (43%) |
| Several days | 10 (18%) | 13 (25%) | 14 (25%) |
| More than half the days | 13 (24%) | 7 (14%) | 8 (14%) |
| Nearly every day | 13 (24%) | 6 (12%) | 10 (18%) |
| Unknown/Missing/Screened negative | 9 | 13 | 8 |
| ^1^ n (%) | | | |

| **Table S11. GAD -7** | | | |
| --- | --- | --- | --- |
|  | **Transgender,**  **N = 64^1^** | **Matched Female,**  **N = 64^1^** | **Matched Male,**  **N = 64^1^** |
| Feeling nervous, anxious, or on edge | | | |
| Not at all | 0 (0%) | 1 (1.8%) | 4 (7.1%) |
| Several days | 7 (12%) | 11 (19%) | 11 (20%) |
| More than half the days | 13 (23%) | 12 (21%) | 11 (20%) |
| Nearly every day | 36 (64%) | 33 (58%) | 30 (54%) |
| Unknown/Missing/Screened negative | 8 | 7 | 8 |
| Not being able stop or control worrying | | | |
| Not at all | 5 (8.9%) | 3 (5.3%) | 4 (7.1%) |
| Several days | 6 (11%) | 13 (23%) | 12 (21%) |
| More than half the days | 14 (25%) | 14 (25%) | 16 (29%) |
| Nearly every day | 31 (55%) | 27 (47%) | 24 (43%) |
| Unknown/Missing/Screened negative | 8 | 7 | 8 |
| Worrying too much about different things | | | |
| Not at all | 4 (7.1%) | 3 (5.3%) | 5 (8.9%) |
| Several days | 7 (12%) | 12 (21%) | 7 (12%) |
| More than half the days | 14 (25%) | 12 (21%) | 20 (36%) |
| Nearly every day | 31 (55%) | 30 (53%) | 24 (43%) |
| Unknown/Missing/Screened negative | 8 | 7 | 8 |
| Trouble relaxing | | | |
| Not at all | 2 (3.6%) | 2 (3.5%) | 6 (11%) |
| Several days | 9 (16%) | 10 (18%) | 8 (14%) |
| More than half the days | 14 (25%) | 16 (28%) | 14 (25%) |
| Nearly every day | 31 (55%) | 29 (51%) | 28 (50%) |
| Unknown/Missing/Screened negative | 8 | 7 | 8 |
| Being so restless that its hard to sit still | | | |
| Not at all | 6 (11%) | 8 (14%) | 12 (21%) |
| Several days | 13 (23%) | 16 (28%) | 11 (20%) |
| More than half the days | 13 (23%) | 11 (19%) | 14 (25%) |
| Nearly every day | 24 (43%) | 22 (39%) | 19 (34%) |
| Unknown/Missing/Screened negative | 8 | 7 | 8 |
| Becoming easily annoyed or irritable | | | |
| Not at all | 1 (1.8%) | 4 (7.0%) | 5 (8.9%) |
| Several days | 14 (25%) | 15 (26%) | 16 (29%) |
| More than half the days | 12 (21%) | 13 (23%) | 14 (25%) |
| Nearly every day | 29 (52%) | 25 (44%) | 21 (38%) |
| Unknown/Missing/Screened negative | 8 | 7 | 8 |
| Feeling afraid as if something awful might happen | | | |
| Not at all | 10 (18%) | 13 (23%) | 9 (16%) |
| Several days | 13 (23%) | 14 (25%) | 12 (21%) |
| More than half the days | 9 (16%) | 8 (14%) | 16 (29%) |
| Nearly every day | 24 (43%) | 22 (39%) | 19 (34%) |
| Unknown/Missing/Screened negative | 8 | 7 | 8 |
| ^1^ n (%) | | | |

| **Table S12. PTSD** | | | |
| --- | --- | --- | --- |
|  | **Transgender,**  **N = 64^1^** | **Matched Female,**  **N = 64^1^** | **Matched Male,**  **N = 64^1^** |
| Repeated, disturbing memories, thoughts or images, of a stressful experience from the past | | | |
| Not at all | 4 (8.2%) | 1 (1.9%) | 6 (11%) |
| A little bit | 3 (6.1%) | 11 (21%) | 13 (24%) |
| Moderately | 9 (18%) | 6 (12%) | 8 (15%) |
| Quite a bit | 9 (18%) | 17 (33%) | 7 (13%) |
| Extremely | 24 (49%) | 17 (33%) | 20 (37%) |
| Unknown/Missing/Screened negative | 15 | 12 | 10 |
| Feeling very upset when something reminded you of a stressful experience from the past | | | |
| Not at all | 3 (6.1%) | 0 (0%) | 6 (11%) |
| A little bit | 4 (8.2%) | 7 (13%) | 9 (17%) |
| Moderately | 10 (20%) | 13 (25%) | 8 (15%) |
| Quite a bit | 8 (16%) | 14 (27%) | 11 (20%) |
| Extremely | 24 (49%) | 18 (35%) | 20 (37%) |
| Unknown/Missing/Screened negative | 15 | 12 | 10 |
| Avoiding activities or situations because they reminded you of a stressful experience from the past | | | |
| Not at all | 7 (14%) | 4 (7.7%) | 11 (20%) |
| A little bit | 5 (10%) | 12 (23%) | 11 (20%) |
| Moderately | 7 (14%) | 6 (12%) | 12 (22%) |
| Quite a bit | 8 (16%) | 13 (25%) | 8 (15%) |
| Extremely | 22 (45%) | 17 (33%) | 12 (22%) |
| Unknown/Missing/Screened negative | 15 | 12 | 10 |
| Feeling distant or cut off from other people | | | |
| Not at all | 3 (6.1%) | 5 (9.6%) | 2 (3.7%) |
| A little bit | 5 (10%) | 4 (7.7%) | 4 (7.4%) |
| Moderately | 4 (8.2%) | 5 (9.6%) | 8 (15%) |
| Quite a bit | 14 (29%) | 12 (23%) | 17 (31%) |
| Extremely | 23 (47%) | 26 (50%) | 23 (43%) |
| Unknown/Missing/Screened negative | 15 | 12 | 10 |
| Feeling irritable or having angry outbursts | | | |
| Not at all | 5 (10%) | 5 (9.6%) | 7 (13%) |
| A little bit | 7 (14%) | 10 (19%) | 13 (24%) |
| Moderately | 7 (14%) | 13 (25%) | 12 (22%) |
| Quite a bit | 9 (18%) | 8 (15%) | 12 (22%) |
| Extremely | 21 (43%) | 16 (31%) | 10 (19%) |
| Unknown/Missing/Screened negative | 15 | 12 | 10 |
| Difficulty concentrating | | | |
| Not at all | 3 (6.1%) | 2 (3.8%) | 5 (9.3%) |
| A little bit | 3 (6.1%) | 8 (15%) | 6 (11%) |
| Moderately | 6 (12%) | 8 (15%) | 11 (20%) |
| Quite a bit | 14 (29%) | 14 (27%) | 14 (26%) |
| Extremely | 23 (47%) | 20 (38%) | 18 (33%) |
| Unknown/Missing/Screened negative | 15 | 12 | 10 |
| ^1^ n (%) | | | |

| **Table S13. Mania screening** | | | |
| --- | --- | --- | --- |
|  | **Transgender,**  **N = 64^1^** | **Matched Female,**  **N = 64^1^** | **Matched Male,**  **N = 64^1^** |
| Happiness or cheerfulness during the last 7 days | | | |
| I did not feel that way more than usual | 5 (56%) | 7 (54%) | 10 (53%) |
| I occasionally felt that way than usual | 2 (22%) | 3 (23%) | 8 (42%) |
| I often that way than usual | 1 (11%) | 1 (7.7%) | 0 (0%) |
| I felt that way most of the time | 1 (11%) | 1 (7.7%) | 1 (5.3%) |
| I that way all of the time | 0 (0%) | 1 (7.7%) | 0 (0%) |
| Unknown/Missing/Screened negative | 55 | 51 | 45 |
| Self-confidence during the last 7 days | | | |
| I did not feel that way more than usual | 5 (56%) | 7 (54%) | 7 (37%) |
| I occasionally felt that way than usual | 3 (33%) | 3 (23%) | 4 (21%) |
| I often that way than usual | 0 (0%) | 2 (15%) | 6 (32%) |
| I felt that way most of the time | 0 (0%) | 0 (0%) | 1 (5.3%) |
| I that way all of the time | 1 (11%) | 1 (7.7%) | 1 (5.3%) |
| Unknown/Missing/Screened negative | 55 | 51 | 45 |
| Less need for sleep during the last 7 days | | | |
| I did not feel that way more than usual | 4 (44%) | 7 (54%) | 10 (53%) |
| I occasionally felt that way than usual | 0 (0%) | 2 (15%) | 4 (21%) |
| I often that way than usual | 2 (22%) | 1 (7.7%) | 1 (5.3%) |
| I felt that way most of the time | 1 (11%) | 0 (0%) | 1 (5.3%) |
| I that way all of the time | 2 (22%) | 3 (23%) | 3 (16%) |
| Unknown/Missing/Screened negative | 55 | 51 | 45 |
| Talkativeness during the last 7 days | | | |
| I did not feel that way more than usual | 3 (33%) | 11 (85%) | 9 (47%) |
| I occasionally felt that way than usual | 3 (33%) | 2 (15%) | 5 (26%) |
| I often that way than usual | 3 (33%) | 0 (0%) | 1 (5.3%) |
| I felt that way most of the time | 0 (0%) | 0 (0%) | 3 (16%) |
| I that way all of the time | 0 (0%) | 0 (0%) | 1 (5.3%) |
| Unknown/Missing/Screened negative | 55 | 51 | 45 |
| Activeness during the last 7 days | | | |
| I did not feel that way more than usual | 5 (56%) | 7 (54%) | 10 (53%) |
| I occasionally felt that way than usual | 3 (33%) | 2 (15%) | 6 (32%) |
| I often that way than usual | 0 (0%) | 1 (7.7%) | 1 (5.3%) |
| I felt that way most of the time | 1 (11%) | 1 (7.7%) | 1 (5.3%) |
| I that way all of the time | 0 (0%) | 2 (15%) | 1 (5.3%) |
| Unknown/Missing/Screened negative | 55 | 51 | 45 |
| ^1^ n (%) | | | |

| **Table S14. PRIME Screening for Psychosis** | | | |
| --- | --- | --- | --- |
|  | **Transgender**,  N = 64^1^ | **Matched Female**,  N = 64^1^ | **Matched Male**,  N = 64^1^ |
| I think I have felt that there are odd or unusual things going on that I can’t explain | | | |
| Definitely disagree | 1 (14%) | 3 (38%) | 1 (10%) |
| Somewhat disagree | 2 (29%) | 0 (0%) | 0 (0%) |
| Slightly disagree | 0 (0%) | 0 (0%) | 1 (10%) |
| Not sure | 1 (14%) | 2 (25%) | 2 (20%) |
| Slightly agree | 1 (14%) | 1 (12%) | 2 (20%) |
| Somewhat agree | 0 (0%) | 0 (0%) | 1 (10%) |
| Definitely agree | 2 (29%) | 2 (25%) | 3 (30%) |
| Unknown/Missing/Screened negative | 57 | 56 | 54 |
| I think I might be able to predict the future | | | |
| Definitely disagree | 3 (43%) | 3 (38%) | 3 (30%) |
| Somewhat disagree | 1 (14%) | 0 (0%) | 0 (0%) |
| Slightly disagree | 0 (0%) | 0 (0%) | 2 (20%) |
| Not sure | 0 (0%) | 1 (12%) | 1 (10%) |
| Slightly agree | 1 (14%) | 2 (25%) | 2 (20%) |
| Somewhat agree | 0 (0%) | 0 (0%) | 1 (10%) |
| Definitely agree | 2 (29%) | 2 (25%) | 1 (10%) |
| Unknown/Missing/Screened negative | 57 | 56 | 54 |
| I may have felt that there could possibly be something interrupting or controlling my thoughts, feelings, or actions | | | |
| Definitely disagree | 1 (14%) | 2 (25%) | 2 (20%) |
| Somewhat disagree | 1 (14%) | 0 (0%) | 1 (10%) |
| Slightly disagree | 0 (0%) | 0 (0%) | 2 (20%) |
| Not sure | 1 (14%) | 3 (38%) | 0 (0%) |
| Slightly agree | 0 (0%) | 0 (0%) | 2 (20%) |
| Somewhat agree | 2 (29%) | 1 (12%) | 1 (10%) |
| Definitely agree | 2 (29%) | 2 (25%) | 2 (20%) |
| Unknown/Missing/Screened negative | 57 | 56 | 54 |
| I have had the experience of doing something differently because of my superstitions | | | |
| Definitely disagree | 0 (0%) | 4 (50%) | 5 (50%) |
| Somewhat disagree | 1 (14%) | 0 (0%) | 0 (0%) |
| Slightly disagree | 0 (0%) | 0 (0%) | 1 (10%) |
| Not sure | 2 (29%) | 2 (25%) | 0 (0%) |
| Slightly agree | 2 (29%) | 1 (12%) | 1 (10%) |
| Somewhat agree | 0 (0%) | 1 (12%) | 1 (10%) |
| Definitely agree | 2 (29%) | 0 (0%) | 2 (20%) |
| Unknown/Missing/Screened negative | 57 | 56 | 54 |
| I think that I may get confused at times whether something I experience or perceive may be real or may be just part of my imagination or dreams | | | |
| Definitely disagree | 0 (0%) | 4 (50%) | 3 (30%) |
| Somewhat disagree | 1 (14%) | 0 (0%) | 0 (0%) |
| Slightly disagree | 1 (14%) | 1 (12%) | 2 (20%) |
| Not sure | 0 (0%) | 1 (12%) | 0 (0%) |
| Slightly agree | 0 (0%) | 0 (0%) | 1 (10%) |
| Somewhat agree | 3 (43%) | 1 (12%) | 2 (20%) |
| Definitely agree | 2 (29%) | 1 (12%) | 2 (20%) |
| Unknown/Missing/Screened negative | 57 | 56 | 54 |
| I have thought that it might be possible that other can read my mind, or that I can read other’s minds | | | |
| Definitely disagree | 3 (43%) | 5 (62%) | 3 (30%) |
| Somewhat disagree | 1 (14%) | 0 (0%) | 0 (0%) |
| Slightly disagree | 1 (14%) | 0 (0%) | 1 (10%) |
| Not sure | 0 (0%) | 1 (12%) | 1 (10%) |
| Slightly agree | 0 (0%) | 0 (0%) | 2 (20%) |
| Somewhat agree | 0 (0%) | 1 (12%) | 2 (20%) |
| Definitely agree | 2 (29%) | 1 (12%) | 1 (10%) |
| Unknown/Missing/Screened negative | 57 | 56 | 54 |
| I wonder if people may be planning to hurt me or even may be about to hurt me | | | |
| Definitely disagree | 1 (14%) | 3 (38%) | 3 (30%) |
| Somewhat disagree | 2 (29%) | 0 (0%) | 0 (0%) |
| Slightly disagree | 0 (0%) | 0 (0%) | 1 (10%) |
| Not sure | 1 (14%) | 2 (25%) | 0 (0%) |
| Slightly agree | 0 (0%) | 0 (0%) | 2 (20%) |
| Somewhat agree | 0 (0%) | 2 (25%) | 1 (10%) |
| Definitely agree | 3 (43%) | 1 (12%) | 3 (30%) |
| Unknown/Missing/Screened negative | 57 | 56 | 54 |
| I believe that I have special natural or supernatural gifts beyond my talents and natural strengths | | | |
| Definitely disagree | 3 (43%) | 5 (62%) | 5 (50%) |
| Somewhat disagree | 0 (0%) | 0 (0%) | 0 (0%) |
| Slightly disagree | 0 (0%) | 0 (0%) | 1 (10%) |
| Not sure | 0 (0%) | 1 (12%) | 1 (10%) |
| Slightly agree | 1 (14%) | 0 (0%) | 1 (10%) |
| Somewhat agree | 1 (14%) | 0 (0%) | 1 (10%) |
| Definitely agree | 2 (29%) | 2 (25%) | 1 (10%) |
| Unknown/Missing/Screened negative | 57 | 56 | 54 |
| I think I might feel like my mind is “playing tricks” on me | | | |
| Definitely disagree | 1 (14%) | 5 (62%) | 4 (40%) |
| Somewhat disagree | 0 (0%) | 0 (0%) | 0 (0%) |
| Slightly disagree | 2 (29%) | 0 (0%) | 1 (10%) |
| Not sure | 1 (14%) | 1 (12%) | 2 (20%) |
| Slightly agree | 0 (0%) | 1 (12%) | 1 (10%) |
| Somewhat agree | 1 (14%) | 0 (0%) | 1 (10%) |
| Definitely agree | 2 (29%) | 1 (12%) | 1 (10%) |
| Unknown/Missing/Screened negative | 57 | 56 | 54 |
| I have had the experience of hearing faint or clear sounds of people or a person mumbling or talking when there is no one near me | | | |
| Definitely disagree | 2 (29%) | 2 (25%) | 4 (40%) |
| Somewhat disagree | 1 (14%) | 0 (0%) | 0 (0%) |
| Slightly disagree | 0 (0%) | 2 (25%) | 1 (10%) |
| Not sure | 1 (14%) | 1 (12%) | 2 (20%) |
| Slightly agree | 0 (0%) | 1 (12%) | 0 (0%) |
| Somewhat agree | 1 (14%) | 0 (0%) | 1 (10%) |
| Definitely agree | 2 (29%) | 2 (25%) | 2 (20%) |
| Unknown/Missing/Screened negative | 57 | 56 | 54 |
| I think I may hear my own thoughts being said out loud | | | |
| Definitely disagree | 3 (43%) | 3 (38%) | 4 (40%) |
| Somewhat disagree | 0 (0%) | 0 (0%) | 0 (0%) |
| Slightly disagree | 0 (0%) | 0 (0%) | 2 (20%) |
| Not sure | 0 (0%) | 2 (25%) | 2 (20%) |
| Slightly agree | 1 (14%) | 1 (12%) | 0 (0%) |
| Somewhat agree | 0 (0%) | 0 (0%) | 1 (10%) |
| Definitely agree | 3 (43%) | 2 (25%) | 1 (10%) |
| Unknown/Missing/Screened negative | 57 | 56 | 54 |
| I have been concerned that I might be “going crazy” | | | |
| Definitely disagree | 2 (29%) | 2 (25%) | 3 (30%) |
| Somewhat disagree | 0 (0%) | 2 (25%) | 0 (0%) |
| Slightly disagree | 1 (14%) | 0 (0%) | 1 (10%) |
| Not sure | 0 (0%) | 2 (25%) | 2 (20%) |
| Slightly agree | 2 (29%) | 0 (0%) | 1 (10%) |
| Somewhat agree | 1 (14%) | 2 (25%) | 1 (10%) |
| Definitely agree | 1 (14%) | 0 (0%) | 2 (20%) |
| Unknown/Missing/Screened negative | 57 | 56 | 54 |
| ^1^ n (%) | | | |

| **Table S15. EDDS Eating Disorder Screening** | | | |
| --- | --- | --- | --- |
|  | **Transgender**,  N = 64^1^ | **Matched Female**,  N = 64^1^ | **Matched Male**,  N = 64^1^ |
| [Since specified time point], did you feel fat? | | | |
| Not at all | 18 (46%) | 20 (40%) | 31 (67%) |
| Slightly | 6 (15%) | 10 (20%) | 4 (8.7%) |
| Moderately | 5 (13%) | 8 (16%) | 8 (17%) |
| Extremely | 10 (26%) | 12 (24%) | 3 (6.5%) |
| Unknown/Missing/Screened negative | 25 | 14 | 18 |
| [Since specified time point], have you had a definite fear that you might gain weight or become fat? | | | |
| Not at all | 15 (38%) | 15 (30%) | 32 (70%) |
| Slightly | 7 (18%) | 15 (30%) | 6 (13%) |
| Moderately | 4 (10%) | 7 (14%) | 5 (11%) |
| Extremely | 13 (33%) | 13 (26%) | 3 (6.5%) |
| Unknown/Missing/Screened negative | 25 | 14 | 18 |
| [Since specified time point], has your weight or shape influenced how you judged yourself as a person? | | | |
| Not at all | 11 (28%) | 16 (32%) | 28 (61%) |
| Slightly | 9 (23%) | 8 (16%) | 8 (17%) |
| Moderately | 3 (7.7%) | 12 (24%) | 5 (11%) |
| Extremely | 16 (41%) | 14 (28%) | 5 (11%) |
| Unknown/Missing/Screened negative | 25 | 14 | 18 |
| [Since specified time point], were there times where you ate what other people would regard as an unusually large amount of food? | | | |
| No | 27 (69%) | 37 (74%) | 33 (72%) |
| Yes | 12 (31%) | 13 (26%) | 13 (28%) |
| Unknown/Missing/Screened negative | 25 | 14 | 18 |
| During the times when you ate an unusually large amount of food, did you experience a loss of control (e.g., felt you couldn’t stop eating or control what or how much you were eating)? | | | |
| No | 3 (25%) | 5 (38%) | 6 (46%) |
| Yes | 9 (75%) | 8 (62%) | 7 (54%) |
| Unknown/Missing/Screened negative | 52 | 51 | 51 |
| How many times per week on average did you eat an unusually large amount of food and experience a loss of control (please enter a number)? | | | |
| 0 | 0 (0%) | 1 (12%) | 0 (0%) |
| 1 | 1 (11%) | 1 (12%) | 0 (0%) |
| 2 | 2 (22%) | 4 (50%) | 0 (0%) |
| 3 | 1 (11%) | 1 (12%) | 1 (14%) |
| 4 | 1 (11%) | 0 (0%) | 0 (0%) |
| 5 | 1 (11%) | 1 (12%) | 2 (29%) |
| 6 | 1 (11%) | 0 (0%) | 2 (29%) |
| 7 | 1 (11%) | 0 (0%) | 2 (29%) |
| 30 | 1 (11%) | 0 (0%) | 0 (0%) |
| Unknown/Missing/Screened negative | 55 | 56 | 57 |
| During these episodes of overeating and loss of control did you eat much more rapidly than normal? | | | |
| No | 3 (33%) | 2 (25%) | 3 (43%) |
| Yes | 6 (67%) | 6 (75%) | 4 (57%) |
| Unknown/Missing/Screened negative | 55 | 56 | 57 |
| During these episodes of overeating and loss of control did you eat until you felt uncomfortably full? | | | |
| No | 1 (11%) | 2 (25%) | 2 (29%) |
| Yes | 8 (89%) | 6 (75%) | 5 (71%) |
| Unknown/Missing/Screened negative | 55 | 56 | 57 |
| During these episodes of overeating and loss of control did you eat a lot of food when you didnt feel physically hungry? | | | |
| No | 2 (22%) | 2 (25%) | 1 (14%) |
| Yes | 7 (78%) | 6 (75%) | 6 (86%) |
| Unknown/Missing/Screened negative | 55 | 56 | 57 |
| During these episodes of overeating and loss of control did you eat alone because you were embarrassed by how much you were overeating? | | | |
| No | 6 (67%) | 4 (50%) | 6 (86%) |
| Yes | 3 (33%) | 4 (50%) | 1 (14%) |
| Unknown/Missing/Screened negative | 55 | 56 | 57 |
| During these episodes of overeating and loss of control did you feel disgusted with yourself, depressed, or very guilty after overeating | | | |
| No | 3 (33%) | 3 (38%) | 5 (71%) |
| Yes | 6 (67%) | 5 (62%) | 2 (29%) |
| Unknown/Missing/Screened negative | 55 | 56 | 57 |
| During these episodes of overeating and loss of control did you feel upset with yourself? | | | |
| No | 3 (33%) | 4 (50%) | 3 (43%) |
| Yes | 6 (67%) | 4 (50%) | 4 (57%) |
| Unknown/Missing/Screened negative | 55 | 56 | 57 |
| How often [in specified time period], did you make yourself vomit | | | |
| 0 | 34 (83%) | 46 (92%) | 46 (100%) |
| 1 | 0 (0%) | 2 (4.0%) | 0 (0%) |
| 2 | 4 (9.8%) | 1 (2.0%) | 0 (0%) |
| 3 | 2 (4.9%) | 0 (0%) | 0 (0%) |
| 7 | 0 (0%) | 1 (2.0%) | 0 (0%) |
| 32 | 1 (2.4%) | 0 (0%) | 0 (0%) |
| Unknown/Missing/Screened negative | 23 | 14 | 18 |
| How often [in specified time period], did you use laxatives or diuretics | | | |
| 0 | 36 (88%) | 48 (96%) | 46 (100%) |
| 2 | 1 (2.4%) | 0 (0%) | 0 (0%) |
| 4 | 2 (4.9%) | 0 (0%) | 0 (0%) |
| 5 | 0 (0%) | 2 (4.0%) | 0 (0%) |
| 6 | 1 (2.4%) | 0 (0%) | 0 (0%) |
| 32 | 1 (2.4%) | 0 (0%) | 0 (0%) |
| Unknown/Missing/Screened negative | 23 | 14 | 18 |
| How often [in specified time period], did you fast (skip at least 2 meals in a row) | 0.0 (0.0, 5.0) | 0.5 (0.0, 4.0) | 0.0 (0.0, 2.0) |
| Unknown/Missing/Screened negative | 23 | 14 | 18 |
| How often [in specified time period], did you engage in more intense exercise to counteract effects of overeating | | | |
| 0 | 32 (78%) | 44 (88%) | 41 (89%) |
| 1 | 3 (7.3%) | 4 (8.0%) | 1 (2.2%) |
| 2 | 0 (0%) | 0 (0%) | 2 (4.3%) |
| 3 | 1 (2.4%) | 2 (4.0%) | 0 (0%) |
| 4 | 1 (2.4%) | 0 (0%) | 0 (0%) |
| 5 | 1 (2.4%) | 0 (0%) | 2 (4.3%) |
| 7 | 1 (2.4%) | 0 (0%) | 0 (0%) |
| 10 | 1 (2.4%) | 0 (0%) | 0 (0%) |
| 32 | 1 (2.4%) | 0 (0%) | 0 (0%) |
| Unknown/Missing/Screened negative | 23 | 14 | 18 |
| How often [in specified time period], how many times did you eat during the night after awakening from sleep or eat a large amount food after your evening meal? | 0.00 (0.00, 1.00) | 0.00 (0.00, 2.00) | 0.00 (0.00, 0.00) |
| Unknown/Missing/Screened negative | 23 | 14 | 18 |
| How often [in specified time period], how much did eating or body image problems impact your relationships, work, school | | | |
| 0 | 22 (56%) | 22 (44%) | 37 (80%) |
| 2 | 8 (21%) | 11 (22%) | 7 (15%) |
| 4 | 4 (10%) | 14 (28%) | 1 (2.2%) |
| 6 | 5 (13%) | 3 (6.0%) | 1 (2.2%) |
| Unknown/Missing/Screened negative | 25 | 14 | 18 |
| ^1^ n (%); Median (IQR) | | | |

| **Table S16. Functioning** | | | |
| --- | --- | --- | --- |
|  | **Transgender,**  **N = 64^1^** | **Matched Female,**  **N = 64^1^** | **Matched Male,**  **N = 64^1^** |
| [In time period], how much difficulty did you have standing for long periods such as 30 minutes? | | | |
| None | 0 (0%) | 4 (67%) | 5 (56%) |
| Mild | 0 (0%) | 0 (0%) | 1 (11%) |
| Moderate | 2 (67%) | 2 (33%) | 1 (11%) |
| Extreme or could not do | 1 (33%) | 0 (0%) | 2 (22%) |
| Unknown/Missing/Screened negative | 61 | 58 | 55 |
| [In time period], how much difficulty did you have taking care of your household responsibilities? | | | |
| None | 0 (0%) | 2 (33%) | 2 (22%) |
| Mild | 0 (0%) | 0 (0%) | 1 (11%) |
| Moderate | 1 (33%) | 3 (50%) | 3 (33%) |
| Severe | 1 (33%) | 1 (17%) | 1 (11%) |
| Extreme or could not do | 1 (33%) | 0 (0%) | 2 (22%) |
| Unknown/Missing/Screened negative | 61 | 58 | 55 |
| [In time period], how much difficulty did you have learning a new task, such as learning how to get to a new place? | | | |
| None | 0 (0%) | 3 (50%) | 4 (44%) |
| Mild | 0 (0%) | 1 (17%) | 0 (0%) |
| Moderate | 1 (33%) | 2 (33%) | 2 (22%) |
| Severe | 1 (33%) | 0 (0%) | 1 (11%) |
| Extreme or could not do | 1 (33%) | 0 (0%) | 2 (22%) |
| Unknown/Missing/Screened negative | 61 | 58 | 55 |
| [In time period], how much difficulty did you have joining in community activities (such as festivities or religious activities) in the same way that anyone else could? | | | |
| None | 0 (0%) | 3 (50%) | 2 (22%) |
| Moderate | 1 (33%) | 3 (50%) | 0 (0%) |
| Severe | 1 (33%) | 0 (0%) | 4 (44%) |
| Extreme or could not do | 1 (33%) | 0 (0%) | 3 (33%) |
| Unknown/Missing/Screened negative | 61 | 58 | 55 |
| [In time period], how much difficulty did you have concentrating on doing something for 10 minutes? | | | |
| None | 0 (0%) | 2 (33%) | 2 (22%) |
| Mild | 0 (0%) | 1 (17%) | 3 (33%) |
| Moderate | 1 (33%) | 3 (50%) | 1 (11%) |
| Severe | 1 (33%) | 0 (0%) | 1 (11%) |
| Extreme or could not do | 1 (33%) | 0 (0%) | 2 (22%) |
| Unknown/Missing/Screened negative | 61 | 58 | 55 |
| [In time period], how much difficulty did you have walking a long distance, such as half a mile? | | | |
| None | 0 (0%) | 3 (50%) | 4 (44%) |
| Mild | 1 (33%) | 1 (17%) | 2 (22%) |
| Moderate | 0 (0%) | 2 (33%) | 0 (0%) |
| Severe | 1 (33%) | 0 (0%) | 1 (11%) |
| Extreme or could not do | 1 (33%) | 0 (0%) | 2 (22%) |
| Unknown/Missing/Screened negative | 61 | 58 | 55 |
| [In time period], how much difficulty did you have washing your whole body? | | | |
| None | 1 (33%) | 2 (33%) | 4 (44%) |
| Mild | 0 (0%) | 1 (17%) | 1 (11%) |
| Moderate | 2 (67%) | 3 (50%) | 0 (0%) |
| Severe | 0 (0%) | 0 (0%) | 3 (33%) |
| Extreme or could not do | 0 (0%) | 0 (0%) | 1 (11%) |
| Unknown/Missing/Screened negative | 61 | 58 | 55 |
| [In time period], how much difficulty did you have getting dressed? | | | |
| None | 2 (67%) | 2 (33%) | 4 (44%) |
| Mild | 0 (0%) | 0 (0%) | 1 (11%) |
| Moderate | 1 (33%) | 3 (50%) | 2 (22%) |
| Severe | 0 (0%) | 1 (17%) | 1 (11%) |
| Extreme or could not do | 0 (0%) | 0 (0%) | 1 (11%) |
| Unknown/Missing/Screened negative | 61 | 58 | 55 |
| [In time period], how much difficulty did you have dealing with people you do not know? | | | |
| None | 0 (0%) | 2 (33%) | 2 (22%) |
| Mild | 1 (33%) | 0 (0%) | 2 (22%) |
| Moderate | 1 (33%) | 3 (50%) | 3 (33%) |
| Severe | 1 (33%) | 1 (17%) | 2 (22%) |
| Unknown/Missing/Screened negative | 61 | 58 | 55 |
| [In time period], how much difficulty did you have maintaining a friendship? | | | |
| None | 0 (0%) | 2 (33%) | 0 (0%) |
| Mild | 0 (0%) | 1 (17%) | 2 (22%) |
| Moderate | 2 (67%) | 3 (50%) | 1 (11%) |
| Severe | 1 (33%) | 0 (0%) | 5 (56%) |
| Extreme or could not do | 0 (0%) | 0 (0%) | 1 (11%) |
| Unknown/Missing/Screened negative | 61 | 58 | 55 |
| [In time period], how much difficulty did you have managing your day-to-day work? | | | |
| None | 0 (0%) | 2 (33%) | 2 (22%) |
| Mild | 0 (0%) | 0 (0%) | 1 (11%) |
| Moderate | 1 (33%) | 3 (50%) | 2 (22%) |
| Severe | 1 (33%) | 1 (17%) | 3 (33%) |
| Extreme or could not do | 1 (33%) | 0 (0%) | 1 (11%) |
| Unknown/Missing/Screened negative | 61 | 58 | 55 |
